# Role of cardiovascular computed tomography parameters and lungs findings in predicting severe COVID-19 patients: a single-centre retrospective study

**DOI:** 10.1186/s43055-022-00910-0

**Published:** 2022-10-17

**Authors:** Mahmoud Mousa, Marwan Matar, Mohammad Matar, Sadi Jaber, Fouad S. Jaber, Yasser Al Ajerami, Amjad Falak, Mohammed Abujazar, Ammar A. Oglat, Hammoda Abu-Odah

**Affiliations:** 1 Department of Radiology, Turkish Friendship Hospital, Gaza Strip, Palestine; 2 Department of Radiology, Al-Shifa Medical Complex, Gaza Strip, Palestine; 3 Department of Radiology, Nasser Medical Complex, Gaza Strip, Palestine; 4 Internal Medicine Department, University of Missouri–Kansas City, Missouri, USA; 5 Department of Medical Imaging, Applied Medical Sciences, Al-Azhar University, Gaza Strip, Palestine; 6 Department of Advanced Material Technologies, Faculty of Material Engineering, Silesian University of Technology (SUT), Gliwice, Poland; 7 Center for Medical Imaging, Uppsala University Hospital, 75185 Uppsala, Sweden; 8 Department of Medical Imaging, Faculty of Applied Medical Sciences, The Hashemite University, Zarqa, 13133 Jordan; 9 School of Nursing, The Hong Kong Polytechnic University, FG 414 a-b, 11 Yuk Choi Rd, Hung Hom, Hong Kong SAR, China

**Keywords:** COVID-19, Chest CT, Cardiovascular, Lung findings, Death predictor

## Abstract

**Background:**

During the coronavirus disease 2019 (COVID-19) pandemic, most patients experienced various respiratory and cardiovascular problems, and their health suddenly deteriorated despite active treatment. Many parameters have been used to assess patient health status. However, few have considered chest computed tomography (CCT) and lung findings to predict patient outcomes. This single-centre retrospective observational study was conducted between December 2020 and March 2021 at the European Gaza Hospital to predict the mortality of COVID-19 patients based on their CCT parameters and lung involvement scores.

**Results:**

A total of 152 patients with severe respiratory symptoms were admitted during the study period, of which 93 (61.2%) improved and 59 (38.8%) died. Deceased patients showed a significantly higher right pulmonary diameter, cardiothoracic ratio, and ground glass with crazy paving opacity (*p* < 0.05). A cardiothoracic ratio ≥ 0.49 was associated with significantly higher mortality risk (*p* < 0.05) and a fourfold higher hazard ratio (*p* < 0.05) compared to < 0.49.

**Conclusions:**

Assessing cardiac indices on CCT could provide prognostic information and guide physicians in patient management and risk stratification.

## Background

The 2019 coronavirus disease (COVID-19) pandemic has negatively influenced the cardiovascular system of patients and posed challenges to the global cardiovascular community [1]. About 40% of COVID‐19 deaths are due to cardiovascular involvement [2], potentially reflecting direct or indirect effects such as myocardial injury, myocarditis, acute coronary syndrome, cardiac arrhythmias, heart failure, cardiogenic shock [3, 4], and pulmonary hypertension [5]. The disease severity and survival rates could be associated with demographic and clinical variables, including age, sex, and comorbidities such as cardiovascular conditions [6–9]. For example, cardiovascular risks such as myocarditis, acute myocardial infarction, and sudden onset of heart failure were noted in previous influenza epidemics, with a substantial increase in morbidity and mortality [10, 11].

There is currently a dearth of research on COVID-19 infection in patients with pulmonary hypertension and cardiovascular index, resulting in poor evidence-based guidance to manage this specific patient population and reliably predict their clinical course [12]. In addition, further research is needed to investigate the cardiovascular parameters as predictors of morbidity and mortality.

The dilatation of the main pulmonary artery is a sign of increasing pulmonary pressure, most often due to increased pulmonary artery resistance. Consequently, a pulmonary artery-to**-**ascending aorta (PA/AA) diameter ratio of > 1 raises suspicion of pulmonary hypertension [13–16] and is associated with an unfavourable prognosis in patients with respiratory diseases [17, 18]. Chest computed tomography (CCT) has high sensitivity and clinical value in COVID-19 diagnosis [19–22]. Many cardiovascular parameters could be assessed with CCT, including the cardiothoracic ratio (CTR), referring to cardiomegaly [23], PA/AA ratio, and inferior vena cava (IVC) dimensions. In addition, CCT helps to assess the severity of lung involvement via calculating the total lung score in all zones (upper, middle, and lower), with higher scores associated with more severe COVID-19 disease [24, 25]. The clinical utility of cardiovascular parameters and lung involvement scores could be important in providing prognostic information and facilitating risk stratification among COVID-19 patients. Therefore, this study predicts the mortality of COVID-19 patients based on CCT cardiovascular parameters and lung involvement scores.

## Methods

### Study design, sample, and population

This retrospective observational study included 152 non-vaccinated COVID-19 patients admitted at the European Gaza Hospital in the Gaza Strip, Palestine. CCT data were collected between December 2020 and March 2021 during their hospitalization.

### Eligibility criteria

#### Inclusion criteria

Patient inclusion criteria were: aged ≥ 18 years, confirmed COVID-19 infection, and availability of non-contrast CCT data.

#### Exclusion criteria

Patient exclusion criteria were a history of pulmonary emboli or oxygen-dependent chronic obstructive pulmonary disease and poor CT image quality due to respiration or cardiac motion or metallic artefacts.

### Image acquisition and analysis

CCT imaging was acquired for each COVID-19 patient using a Philips-Brilliance 64-slice CT Scanner in the supine position during end-inspiration. The CCT protocol used included scanning parameters such as a 1.5-s scan time, 0.60 mm × 64-detector array, a pitch of 1, table speed of 50 mm/rotation, 200 mAs, 120 kVp, and 5 mm slice thickness. A 1 mm reconstruction interval was used for sagittal and coronal image reconstruction.

CCT data were extracted from the Digital Imaging and Communications in Medicine network system. Two expert consultant radiologists with at least 10 years of experience independently interpreted the images, and a final decision was reached by consensus. In cases of disagreement, the opinion of a third arbitrator consultant radiologist was taken. The images were viewed in axial, sagittal, and coronal planes.

The radiological findings were defined as ground-glass opacification (GGO), consolidation, reticular, crazy-paving pattern, and mixed involvement patterns. Other associated features were observed, including pleural and pericardial effusion, pneumothorax, airway thickening/dilatation, pulmonary vessel dilatation, air bronchogram, traction bronchiectasis, and lymph nodes > 1 cm. The lung opacity distribution was divided into peripheral, central, and peripheral with central. Similarly, lung opacity locations were defined as the upper, middle, and lower lung zones.

Abnormal lung opacities in CCT images were scored by assessing all zonal lung involvements. Independently, each lung consists of three zones: the upper zone, which is located above the carina; the middle zone, which is located between the carina and the inferior pulmonary vein; and the lower zone, which is located below the inferior pulmonary vein. The percentage of severity scores for each lung involvement was calculated via the next classification (score 0: no involvement/normal lung zone; score 1: < 25%; score 2: 26–50%; score 3: 51–75%; score 4: > 75%). The overall lung score (maximum score = 24) was calculated by summing the scores of all three zones [9, 26, 27].

**Fig. 1:**
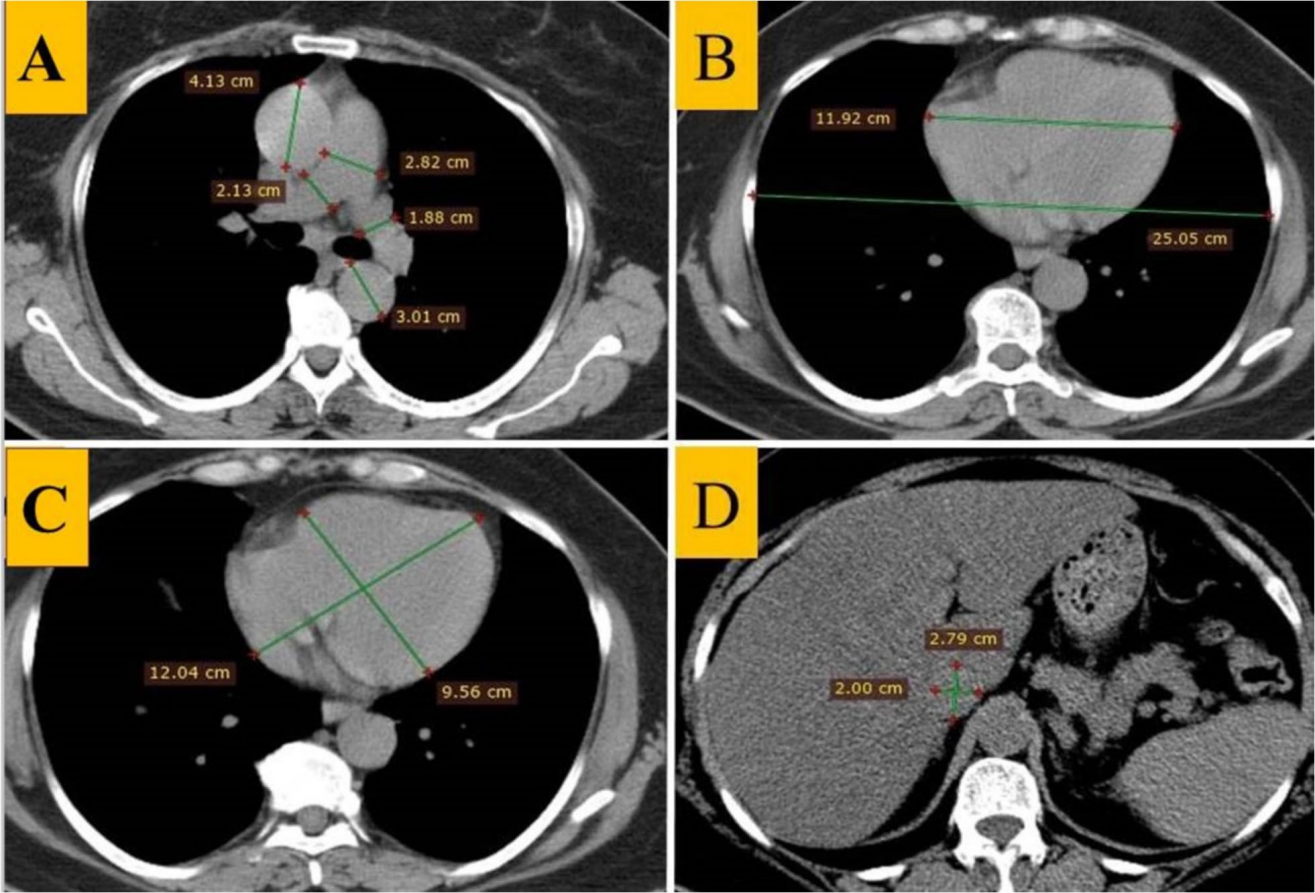
Methods for measuring the CCT cardiovascular parameters. **A** Measurements of the diameters of the pulmonary trunk, right, and left pulmonary, ascending, and descending aorta, and calculation of the PA/AA ratio. **B** Measurements of the heart width and thoracic interval diameters to calculate the CTR. **C** Measurements of the long and short heart axis and calculation of the long/short axis heart ratio. **D** Measurements of the transverse and anterior–posterior IVC diameter and calculation of the Tran/AP ratio

Cardiovascular parameters were assessed by measuring the vessel’s diameter through CCT axial images with mediastinal windows. In addition, we focused on measurements of the main, right, and left pulmonary artery diameters, ascending and descending aorta, and PA/AA ratio. The PA/AA diameter ratio was measured at the main pulmonary trunk bifurcation level (Fig. 1A). Pulmonary trunk enlargement was defined as a PA/AA > 1 to identify patients at increased risk for exacerbations, with a higher ratio correlating with higher pulmonary artery pressure [28]. Then, heart width and thoracic interval diameters were measured to calculate the CTR. CTR was measured through axial images, typically at the diaphragmatic apex level and defined as the greatest transverse cardiac diameter from outer to outer myocardium divided by the greatest transverse thoracic diameter from inner to the inner chest wall (Fig. 1B) [29]. Normal CTR measurements are between 0.42 and 0.49. A CTR < 0.42 is usually considered pathologic, while a CTR ≥ 0.49 is considered a sign of cardiomegaly [29]. Additionally, the long and short axes of the heart were measured at the heart apex level (Fig. 1C) [30], and the long/short axis ratio was calculated. In addition, the transverse and anterior–posterior diameter of the inferior vena cava (IVC) was measured below the diaphragm, and the transverse to anterior–posterior (Tran/AP) ratio was calculated (Fig. 1D) [31, 32]. Measuring transverse diameter helps in better for elucidating the anatomical structure of blood vessel diameter [9, 33, 34]. Determining the maximal diameter of vessels from edge to edge is straightforward [9, 34]. Using only one axis at the level of pulmonary bifurcation to serve as a constant landmark in all cases.

### Statistical analysis

Data were analysed using the Statistical Package of Social Sciences (SPSS; v.25). After the homogenous normality test, the parametric variables with descriptive statistics were used as frequencies, percentages, and mean ± standard deviation (SD). Independent sample t, Chi-square, and Fisher’s Exact tests were used to compare groups. A Cox regression survival analysis was performed to investigate the potential predictors of mortality. A binary logistic regression analysis was performed to identify the mortality odds ratio. A *p*-value of less than 0.05 was considered statistically significant.

## Results

### Clinical and laboratory data characteristics

The 152 patients were divided into two groups: recovered (*n* = 93) and deceased (*n* = 59). The mean age in the recovered group (58.7 ± 15.38) was lower than in the deceased group (64.19 ± 12.17). Severe illnesses were significantly more frequent in the recovered group (*n* = 83; 89.2%), while critical conditions were significantly more frequent in the deceased group (*n* = 51; 86.4%).

The recovered group had more frequent ischemic heart disease and hypertension and lower interstitial lung disease and morbid obesity compared to the deceased group. Laboratory findings showed lower D-dimer, ferritin, and urea levels in the recovered group than in the decreased group. The clinical characteristics and laboratory data are summarized in Table 1.

**Table 1:** Clinical and laboratory data characteristics for COVID19 patients

Variables	Patient outcomes (*n* = 152)		*P*-value
	Recovered (*n* = 93)	Deceased (*n* = 59)	
*Age*			
Mean ± (SD)	58.7 ± (15.38)	64.19 ± (12.17)	0.022*
*Age groups*			
40 y and Less	16 (17.2%)	2 (3.4%)	0.007*
41–50 y	9 (9.7%)	5 (8.5%)	
51–60 y	25 (26.9%)	13 (22%)	
61–70 y	24 (25.8%)	22 (37.3%)	
71–80 y	13 (14%)	13 (22%)	
81–90 y	6 (6.5%)	4 (6.8%)	
*Gender*			
Male	41 (44.1%)	28 (47.5%)	0.405
Female	52 (55.9%)	31 (52.5%)	
*Hospitalization period*			
Mean ± (SD)	12.97 ± (8.45)	12.51 ± (6.84)	0.726
*COVID patient’s status*			
Severe illness	83 (89.2%)	8 (13.6%)	0.0001*
Critical illness	10 (10.8%)	51 (86.4%)	
*Morbidity factors*			
Chronic heart disease	29 (31.2%)	24 (40.7%)	0.068
Ischemic heart disease	21 (22.6%)	8 (13.6%)	0.019*
Congestive heart failure disease	2 (2.2%)	5 (8.5%)	
Ischemic and congestive heart disease	7 (7.5%)	11 (18.6%)	
Diabetes mellitus	52 (55.9%)	39 (66.1%)	0.063
Hypertension	57 (61.3%)	47 (79.7%)	0.008*
Liver disease	2 (2.2%)	3 (5.1%)	0.22
Interstitial lung disease	8 (8.6%)	14 (23.7%)	0.008*
Chronic kidney disease	8 (8.6%)	9 (15.3%)	0.093
Cancer	1 (1.1%)	1 (1.7%)	0.478
Morbid obesity	21 (22.6%)	4 (6.8%)	0.006*
*COVID-19 symptoms*			
Fever	67 (72%)	53 (89.8%)	0.005*
Cough	88 (94.6%)	53 (89.8%)	0.135
Shortness of breathing	84 (90.3%)	59 (100%)	0.010*
Headache	54 (58.1%)	32 (54.2%)	0.12
Loss of smell and taste	51 (54.8%)	23 (39%)	0.022*
Diarrhoea	54 (58.1%)	34 (57.6%)	0.134
Nausea& vomiting	31 (33.3%)	22 (37.3%)	0.122
Abdominal pain	26 (28%)	13 (22%)	0.11
Chest pain	47 (50.5%)	41 (69.5%)	0.009*
*Laboratory tests finding*			
WBC	11.79 ± (17.10)	13.11 ± (29.88)	0.729
Platelets	271.37 ± (110.68)	270.44 ± (137.91)	0.964
CRP	8.23 ± (2.54)	5.89 ± (1.89)	0.0001*
D-dimer	1.85 ± (1.24)	4.71 ± (1.73)	0.0001*
Ferritin	431.37 ± (340.52)	795.14 ± (414.07)	0.0001*
Urea	66.66 ± (43.57)	83.51 ± (53.69)	0.045*
Creatinine	1.09 ± (0.98)	1.37 ± (1.1)	0.11
HB	12.11 ± (1.82)	11.34 ± (1.79)	0.012*
Glucose random	195.33 ± (140.64)	232.19 ± (134.3)	0.108

^*^Statistically significant

### Association between CCT findings and patient outcome

The relationship between CCT radiological findings and the two outcome groups is reported in Table 2. The GGO and crazy paving opacity patterns were more frequent in the deceased group than in the recovered group (*p* = 0.001 and *p* = 0.0001, respectively). Consolidation, sup-plural line, reticular and nodular opacities patterns did not differ significantly between groups.

**Table 2:** Radiological findings of lung CT of COVID-19 patients

Variables	Patient outcomes (*n* = 152)		*P*-value
	Recovered (*n* = 93)	Deceased (*n* = 59)	
Predominant patterns involvement			
*Ground-glass opacity*			
Yes	65 (69.9%)	55 (93.2%)	0.001**
No	28 (30.1%)	4 (6.8%)	
*Consolidation opacity*			
Yes	48 (51.6%)	37 (62.7%)	0.12
No	45 (48.4%)	22 (37.3%)	
*Crazy paving opacity*			
Yes	24 (25.8%)	42 (71.2%)	0.0001**
No	69 (74.2%)	17 (28.8%)	
*Sup-plural line opacity*			
Yes	57 (61.3%)	42 (71.2%)	0.227
No	36 (38.7%)	17 (28.8%)	
*Reticular opacity*			
yes	68 (73.1%)	44 (74.6%)	0.853
No	25 (26.9%)	15 (25.4%)	
Nodular opacity			
Yes	41 (44.1%)	27 (45.8%)	0.868
No	52 (55.9%)	32 (54.2%)	
COVID_19 radiological signs			
Halo sign			
Yes	35 (37.6%)	24 (40.7%)	0.735
No	58 (62.4%)	35 (59.3%)	
*Revers Halo sign*			
Yes	13 (14%)	13 (22%)	0.269
No	80 (86%)	46 (78%)	
*Vascular dilatation around lesions*			
Yes	34 (36.6%)	42 (71.2%)	0.00001*
No	59 (63.4%)	17 (28.8%)	
*Air way wall thickness*			
Yes	41 (44.1%)	42 (71.2%)	0.001*
No	52 (55.9%)	17 (28.8%)	
*Interstitial Septa thickness*			
Yes	67 (72%)	51 (86.4%)	0.046*
No	26 (28%)	8 (13.6%)	
*Bronchiectasis*			
Yes	20 (21.5%)	33 (55.9%)	0.00001*
No	73 (78.5%)	26 (44.1%)	
*Honeycombing*			
Yes	2 (2.2%)	12 (20.3%)	0.0002*
No	91 (97.8%)	47 (79.7%)	
*Cavitation*			
Yes	2 (2.2%)	3 (5.1%)	0.377
No	91 (97.8%)	56 (94.9%)	
Opacities distribution extension			
*Peripheral*			
Yes	88 (94.6%)	58 (98.3%)	0.406
No	5 (5.4%)	1 (1.7%)	
*Central perihilar*			
Yes	48 (51.6%)	47 (79.7%)	0.0005*
No	45 (48.4%)	12 (20.3%)	
*Central and peripheral*			
Yes	33 (35.5%)	44 (74.6%)	0.00003*
No	60 (64.5%)	15 (25.4%)	
*Unilateral lung involvement*			
Yes	7 (7.5%)	0 (0%)	0.043*
No	86 (92.5%)	59 (100%)	
*Bilateral lung involvement*			
Yes	85 (91.4%)	59 (100%)	0.023*
No	8 (8.6%)	0 (0%)	
Other radiological features			
*Pleural effusion*			
Yes	16 (17.2%)	17 (28.8%)	0.108
No	77 (82.8%)	42 (71.2%)	
*Pneumothorax*			
Yes	0 (0)%	30 (5.1%)	0.57f
No	93 (100%)	56 (94.9)%	
*Pericardial effusion*			
Yes	5 (5.4%)	3 (5.1%)	0.624
No	88 (94.6%)	56 (94.9%)	
Cardiovascular calcification			
*Pulmonary branch*			
Yes	2 (2.2%)	4 (6.8%)	0.208
No	91 (97.8%)	55 (93.2%)	
*Ascending Aorta calcification*			
yes	30 (32.3%)	22 (37.3%)	0.599
No	63 (67.7%)	37 (62.7%)	
*Descending Aorta calcification*			
yes	30 (32.3%)	26 (44.1%)	0.169
No	63 (67.7%)	33 (55.9%)	
*Coronary calcification*			
yes	37 (39.8%)	26 (44.1%)	0.616
No	56 (60.2%)	33 (55.9%)	

* Statistical significance in Chi-Square test

** Statistical significant in Fisher’s Exact test

CCT-specific radiological COVID-19 signs of vascular dilatation around lesions, airway wall thickness, interstitial septa thickness, bronchiectasis, and honeycombing were more frequent in the deceased group compared to the recovered group (*p* < 0.0001, *p* = 0.001, *p* = 0.046, *p* = 0.0001, and *p* = 0.0002, respectively).

Both central perihilar and central and peripheral sites were significantly increased in the deceased group compared to the recovered group (*p* = 0.005 and *p* = 3.00 × 10^−5^, respectively). While unilateral lung involvement was significantly increased in the recovered group (*p* = 0.043), bilateral lung involvement was significantly increased in the deceased group (*p* = 0.023). The main pulmonary branch, ascending aorta, descending aorta, and coronary artery calcification did not differ significantly between groups.

### Association between cardiovascular CCT-parameters and patient outcome

The right pulmonary diameter was significantly increased in the deceased group compared to the recovered group (*p* = 0.001). The PA/AA ratio showed a nonsignificant increase in the deceased group compared to the recovered group. In addition, heart width diameter (*p* = 0.0001) and CTR (*p* = 0.001) were significantly increased in the deceased group (14.12 ± 2.51 cm and 0.60 ± 0.1, respectively) compared to the recovered group (12.64 ± 1.8 cm and 0.54 ± 0.11, respectively; Table 3).

**Table 3:** Cardiovascular CT-Parameters of COVID-19 patients

Cardiovascular CT-parameters	Patient Outcomes (*n* = 152)		*P*-value
	Recovered (*n* = 93)	Deceased (*n* = 59)	
Main pulmonary diameter	2.91 ± (0.41)	3.1 ± (0.36)	0.062
Right pulmonary diameter	2.18 ± (0.41)	2.40 ± (0.47)	0.001*
Left pulmonary diameter	2.10 ± (0.47)	2.19 ± (0.41)	0.212
Ascending aorta diameter	3.39 ± (0.46)	3.47 ± (0.39)	0.257
Descending aorta diameter	2.70 ± (0.40)	2.78 ± (0.39)	0.221
Pulmonary/Asc. Aorta ratio (PA/AA)	0.86 ± (0.18)	0.87 ± (0.10)	0.421
Heart width	12.64 ± (1.8)	14.12 ± (2.51)	0.0001*
Thoracic intervals	23.5 ± (2.74)	23.4 ± (2.71)	0.875
Cardiothoracic ratio (CTR)	0.54 ± (0.11)	0.60 ± (0.1)	0.0001*
Long_Axis_heart	12.37 ± (1.88)	12.67 ± (1.89)	0.329
Short_Axis_heart	9.95 ± (1.63)	9.95 ± (1.63)	0.484
Long /Short axis ratio	1.28 ± (0.17)	1.26 ± (0.17)	0.783
Trans_IVC diameter	2.47 ± (0.59)	2.42 ± (0.72)	0.151
AP_IVC-diameter	1.77 ± (0.64)	1.59 ± (0.73)	0.074
Tran/AP-IVC ratio	1.46 ± (0.44)	1.46 ± (0.46)	0.592

* Statistical significant

**Fig. 2:**
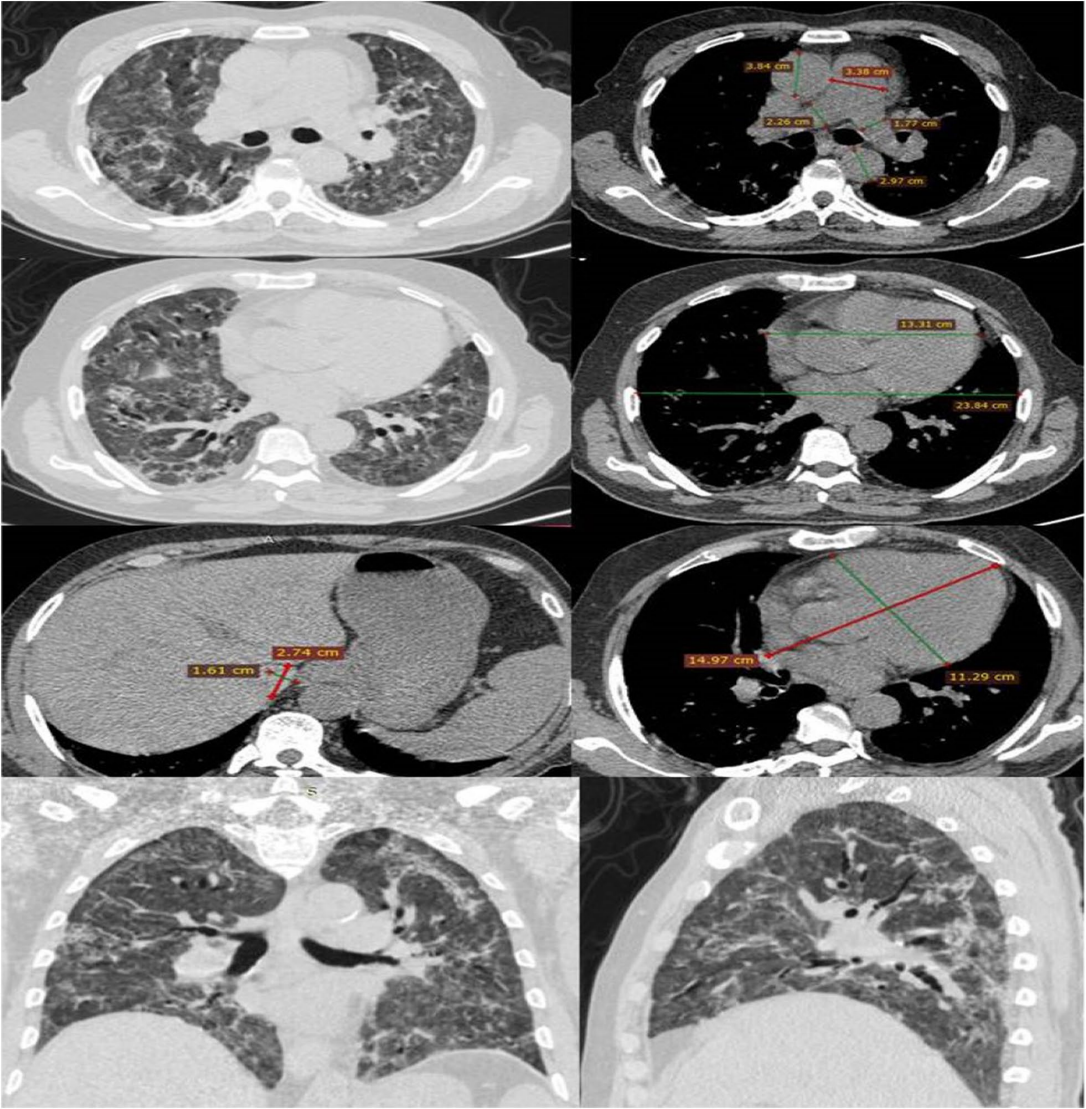
Illustration of the cardiovascular CCT parameters and lung radiological findings in a deceased critical COVID-19 patient. This figure relates to a 58-year-old deceased COVID-19 male patient who suffered from critical symptoms. The predominant lung patterns are GGO and crazy paving opacity bilaterally. This patient has an overall CCT lung involvement score of 24. In addition, it should be noted that cardiomegaly (CTR > 0.49) is present, the PA/AA ratio is ≤ 1, and the right pulmonary artery diameter is greater than the left

In contrast, the long and short axis heart measurements and their ratio did not differ significantly between groups. Similarly, the transverse IVC diameter, anteroposterior IVC diameter, and transverse/anteroposterior IVC ratio did not differ significantly between groups (Fig. 2).

### Odds and hazard ratios of death based on lungs involvement scores and cardiovascular CCT-parameters

Our results show the effects of lung involvement scores and cardiovascular CCT parameters on COVID-19 mortality (Table 4). Lung involvement scores in the right upper, middle, and lower zones and total right scores were significantly increased in the decreased group compared to the recovered group (*p* = 0.0001 in all comparisons). Similarly, the left upper, middle, and lower zones and total left scores were significantly increased in the deceased group compared to the recovered group (*p* = 0.0001 in all comparisons).

**Table 4:** Characteristics of mortality prediction and hazard ratio derived from the lung involvement scores and cardiovascular CT-Parameters measurements

Lung involvement scores	Patent outcome		Logistic model		Cox model	
	Recover (*n* = 93)	Deceased (*n* = 59)	*P*-value	Odd ratio	*P*-value	Hazard ratio
				OR (95% CI)		HR (95% CI)
*Right lung*						
Upper zone score	1 ± (1.5)	3 ± (2)	0.0001*	2.584 (1.83–3.63)	0.002*	1.43 (1.14–1.80)
Middle zone score	2 ± (2)	3.5 ± (1)	0.0001*	4.607 (2.75–7.71)	0.0001*	2.06 (1.48–2.86)
Lower zone score	2.5 ± (2)	4 ± (1)	0.0001*	3.316 (2.12–5.17)	0.001*	1.75 (1.25–2.43)
Total score	6 ± (4)	10 ± (4)	0.0001*	1.761 (1.46–2.11)	0.0001*	1.27 (1.14–1.42)
*Left lung*						
Upper zone score	1 ± (1)	2 ± (2)	0.0001*	2.82 (1.93–4.13)	0.001*	1.46 (1.17–1.83)
Middle zone score	2 ± (1.5)	3.5 ± (1)	0.0001*	7.65 (4.09–14.33)	0.0001*	2.391 (1.69–3.37)
Lower zone score	2 ± (2)	4 ± (1)	0.0001*	4.50 (2.72–7.44)	0.0001*	2.029 (1.45–2.82)
Total score	5.5 ± (3.5)	9 ± (3.5)	0.0001*	2.16 (1.70–2.74)	0.0001*	1.32 (1.18–1.47)
Overall lungs score	12 ± (7)	20 ± (7)	0.0001*	1.46(1.30–1.65)	0.0001*	1.15 (1.09–1.22)
*PA/aorta ratio*			0.484	1.493 (0.48–4.58)	0.863	1.073 (0.48–2.37)
≤ 1.0 (Ref)	83 (89.2%)	52 (88.1%)				
> 1.0	10 (10.8%)	7 (11.9%)				
*CTR*			0.003*	9.877 (2.20–44.24)	0.039*	4.444 (1078–18.32)
< 0.49 (Ref)	23 (24.7%)	2 (3.4%)				
≥ 0.49	70 (75.3%)	57 (96.6%)				

In addition, lung involvement scores in the right upper, middle, and lower zones and total right scores were significantly associated with the hazard ratio (HRs) for death (*p* = 0.002, *p* = 0.0001, *p* = 0.001, and *p* = 0.0001, respectively). Moreover, the left upper, middle, and lower zones and total left scores were significantly associated with the HR for death (*p* = 0.001, *p* = 0.0001, *p* = 0.0001, and *p* = 0.0001, respectively). Overall, both lung scores had significantly increased odds ratio (*p* = 0.0001) and HR (*p* = 0.0001) for death.

In the recovered group, 83 patients (89.2%) had PA/A < 1 and 10 patients (10.8%) who PA/A > 1. In contrast, in the deceased group, 52 patients (88.1%) patients had PA/A < 1 and 7 patients (11.9%) had PA/A > 1. Logistic and Cox regression models did not show a PA/A ratio > 1 to be a predictor of death from COVID-19.

In the recovered group, 23 patients (24.7%) had a CTR < 0.49 and 70 patients (75.3%) had a CTR ≥ 0.49. In contrast, in the deceased group, 2 patients (3.4%) had a CTR < 0.49 and 57 patients (96.6%) had a CTR ≥ 0.49. The logistic and Cox regression models identified CTR ≥ 0.49 as a predictor of death from COVID-19 (Table 4).

**Fig. 3:**
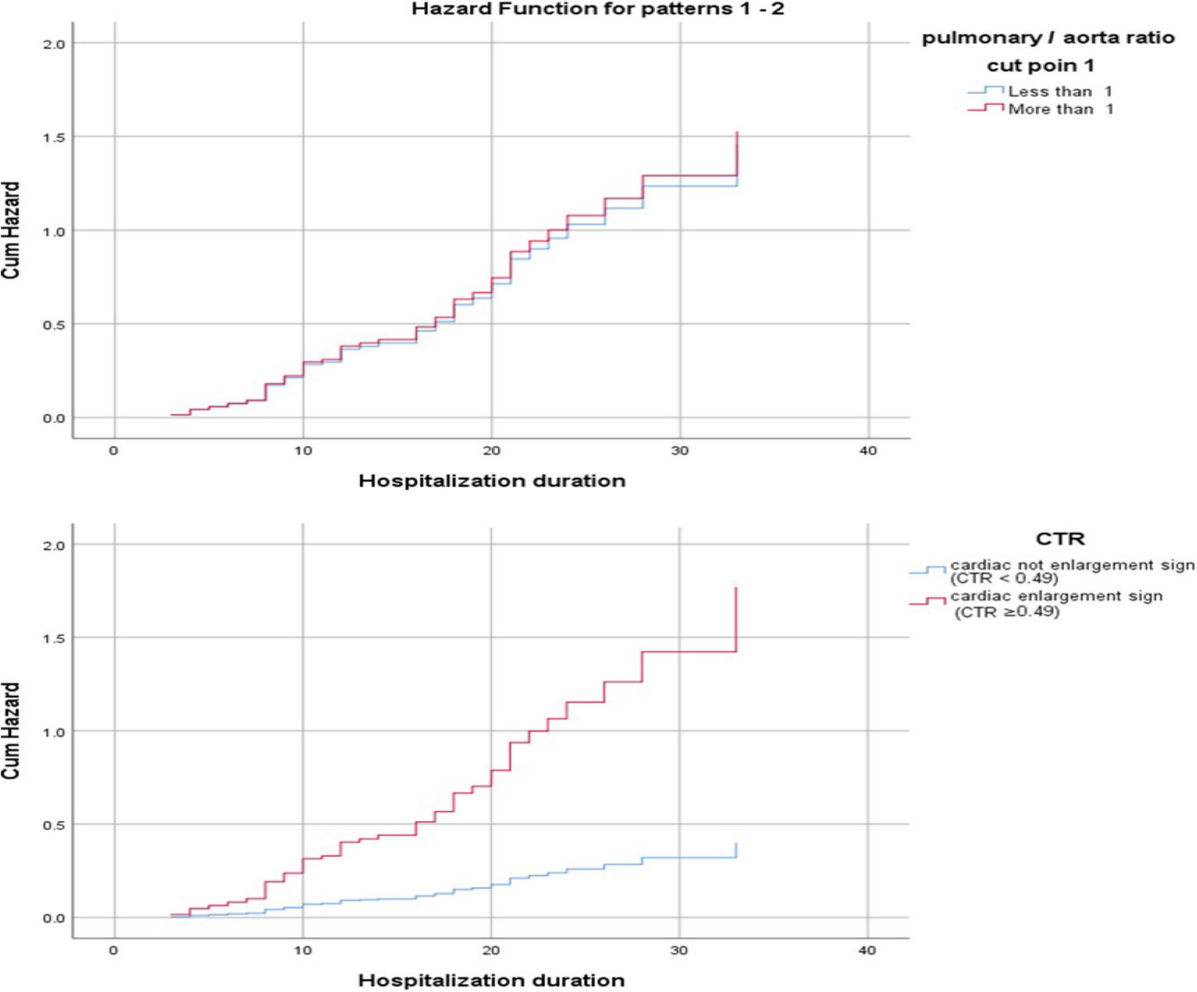
Cumulative HR function for death in COVID-19 patients according to the pulmonary aorta and cardiothoracic ratios. The outcome was defined as death or recovery, and length of hospitalization was considered the time to outcome in the Cox regression analysis

Based on the CTR analysis, the odds ratio for death with a CTR ≥ 0.49 was 9.8-fold higher than with a CTR < 0.49. In addition, a CTR ≥ 0.49 was a significant predictor of the HR for death, which was 4.4-fold higher with a CTR ≥ 0.49 than a CTR < 0.49. The cumulative HR for the pulmonary-aorta and cardiothoracic ratios is illustrated in Fig. 3.

## Discussion

This study adds to the body of knowledge about the prediction of the mortality of COVID-19 patients based on CCT and lung involvement. Our findings show that CCT provides prognostic information that can guide patient management and risk stratification. The CTR was significantly higher in severely hospitalized COVID-19 patients and an independent predictor of COVID-19 mortality. The PA/AA ratio and IVC dimensions were not found to be predictive of COVID-19 patient mortality.

Increased CTR has been associated with a higher risk of adverse cardiovascular events [35]. Our study found that increased cardiomegaly (CTR ≥ 0.49) is frequent in hospitalized COVID-19 patients and strongly predicts their mortality. This finding is similar to a previous study that reported increased CTRs in 76% of patients who eventually died [10]. Our results also indicated that enlarged main pulmonary trunk diameter has high specificity and positive predictive value for the diagnosis of pulmonary hypertension and increased mortality risk. An Italian study reported that an enlarged main pulmonary artery diameter (≥ 31 mm) on the admitting CCT is an independent predictor of mortality in COVID-19 patients [36]. However, another study reported no significant relationship between right and left pulmonary artery diameter and mortality among COVID 19 patients [37].

A PA/AA ratio > 1 has previously been proposed as a biomarker for pulmonary hypertension [38]. Some studies have shown pulmonary embolism, including microembolism, to be one of the causes of severe COVID-19 cases [39, 40]. There has been inconsistency among studies on the increase in PA/AA ratio as a predictor of mortality. In this study, we did not find a significant increase in the odds ratio of COVID-19 mortality in patients with a PA/AA ratio > 1. This finding is similar to another study [9] that found a PA/AA ratio > 1 associated with substantial lung involvement but a nonsignificant increase in the risk of death. In addition, a recent study found that increased pulmonary artery diameter in admitted COVID-19 patients was associated with death [41].

IVC dimensions have been previously suggested as helpful predictive markers of cardiac events and survival [42–45]. However, we could not find an increased risk of death in patients with dilated IVC or an increased long/short heart axis ratio. This finding is supported by another study [9] that reported no increased death among patients with dilated IVC. Moreover, the increase in lung involvement scores on CCT has been associated with the clinical severity and prognosis of COVID-19 patients [46, 47]. Our findings support this since greater lung involvement leads to a significant increase in the odds and risk of death from COVID-19. In addition, deceased patients had much greater involvement in all three zones (upper, middle, and lower), with the middle zone being the most involved with the highest odds ratio for death. We attributed this to its closeness to the main bronchus branches, which have the largest infection burden.

Our use of a cross-sectional design is one limitation of this study, making it difficult to confirm the accuracy of our results. Further comparative research, such as matched control studies, is needed to confirm and extend our findings.

## Conclusions

Our findings indicate that the CTR is significantly higher in hospitalized patients with severe COVID-19 and is an independent predictor of COVID-19 mortality. Moreover, they suggest that assessing cardiac indices on CCT could provide prognostic information that can guide physicians in patient management and risk stratification.

## Data Availability

The datasets used and/or analysed during the current study are available from the first and corresponding authors on reasonable request.

## Abbreviations

CT: Computed tomography
CCT: Chest CT
COVID-19: 2019 Coronavirus disease
CTR: Cardiothoracic ratio
PA/AA: Pulmonary artery diameter, pulmonary artery-to-ascending aorta
IVC: Inferior vena cava
RT-PCR: Reverse transcriptase-polymerase chain reaction
GGO: Ground-glass opacification
